# Transplantation of hUC-MSCs seeded collagen scaffolds reduces scar formation and promotes functional recovery in canines with chronic spinal cord injury

**DOI:** 10.1038/srep43559

**Published:** 2017-03-06

**Authors:** Xing Li, Jun Tan, Zhifeng Xiao, Yannan Zhao, Sufang Han, Dingyang Liu, Wen Yin, Jing Li, Juan Li, Siyi Wanggou, Bing Chen, Caiping Ren, Xingjun Jiang, Jianwu Dai

**Affiliations:** 1 State Key Laboratory of Molecular Developmental Biology, Institute of Genetics and Developmental Biology, Chinese Academy of Sciences, Beijing 100101, China; 2 Department of Neurosurgery, Xiangya Hospital, Central South University, 87 Xiangya Road, Changsha 410008, Hunan Province, China; 3 Cancer Research Institute, Collaborative Innovation Center for Cancer Medicine, School of Basic Medical Science, Central South University, Changsha 410008, China

## Abstract

Spinal cord injury (SCI) can lead to locomotor deficits, and the repair of chronic SCI is considered one of the most challenging clinical problems. Although extensive studies have evaluated treatments for acute SCI in small animals, comparatively fewer studies have been conducted on large-animal SCI in the chronic phase, which is more clinically relevant. Here, we used a collagen-based biomaterial, named the NeuroRegen scaffold, loaded with human umbilical cord-derived mesenchymal stem cells (hUC-MSCs) in a canine chronic SCI model. To generate chronic SCI, the T8 spinal cord segment was removed by complete transection of the spinal cord. Two months later, glial scar tissue was removed and a NeuroRegen scaffold was transplanted into the lesion area. Functionalized NeuroRegen scaffold implantation promoted both locomotor recovery and endogenous neurogenesis in the lesion area. Moreover, some newly generated neurons successfully matured into 5-HT-positive neurons at 1 year post-injury. In addition, many regenerated axon fibers in the lesion area exhibited remyelination and synapse formation at 1 year post-injury in the functionalized NeuroRegen scaffold group. In conclusion, the NeuroRegen scaffold functionalized with hUC-MSCs is a promising potential therapeutic approach to chronic SCI that promotes neuronal regeneration, reduces glial scar formation, and ultimately improves locomotor recovery.

Spinal cord injury (SCI) leading to permanent loss of sensation and voluntary movement below the lesion level is one of the most challenging clinical problems^1^. To date, therapies for SCI are largely ineffective^2^. The main challenges for SCI recovery include the lack of a necessary mechanical scaffold to guide and support axonal regeneration, the presence of various myelin or reactive glia-derived inhibitors of axonal growth, and the absence of sufficient neurotrophic stimulation and growth factor-mediated neuroprotection^3^,^4^,^5^. Specifically, in the chronic phase of SCI, continued demyelination in white matter results in the production of myelin-associated inhibitors (MAIs) such as Nogo, myelin-associated glycoprotein, and oligodendrocyte myelin glycoprotein, which impede axonal regeneration and neurite outgrowth^6^. In addition, chondroitin sulfate proteoglycans (CSPGs) secreted by reactive astrocytes that eventually form glial scars represent another major inhibitor of functional recovery. Treatments specifically targeting MAIs and CSPGs have shown clinical potential for promoting SCI recovery^7^,^8^. However, no current clinical strategy employs these mechanisms to support recovery after SCI. In addition to an adverse microenvironment formed in the lesion site, another challenge for SCI repair is that long-term lower limb paralysis and a lack of specific rehabilitation strategies collectively lead to muscle atrophy and joint dysfunction. Thus, specific rehabilitation strategies may be another important element for effective axonal regeneration and locomotor recovery after SCI^9^.

A number of preclinical studies have demonstrated that mesenchymal stem cell (MSC) therapy holds great promise for the treatment of central nervous system (CNS) injury; administration of MSCs after traumatic brain injury or spinal cord injury reduces secondary neuronal loss and improves functional recovery in animal models of injury^10^,^11^,^12^,^13^,^14^,^15^,^16^,^17^. Among various sources of mesenchymal stem cells, human umbilical cord mesenchymal stem cell (hUC-MSC) retrieval is a highly desirable method for obtaining cells: the collection method is painless, the procedure is minimally controversial in terms of ethics, and the resultant cells exhibit fast self-renewal characteristics^18^. hUC-MSCs also express low levels of major histocompatibility complex class II and co-stimulatory molecules, making them immune-privileged cells for clinical application^19^. Furthermore, hUC-MSCs are suitable autologous or allogeneic agents for the treatment of SCI based on their ability to alter immune cell function and immune responses by regulating the activation and proliferation immune cells (e.g., T-cells and B-cells) and natural killer cells^20^,^21^ that are abundant after SCI. Finally, MSCs exhibit other useful properties for SCI repair including anti-inflammatory, neurotrophic, and angiogenic effects^15^.

Collagen is widely used in tissue engineering owing to its high abundance, low antigenicity, excellent biocompatibility, and biodegradability^22^,^23^,^24^. Collagen has been widely used in scaffolds and gels in nervous tissue regeneration^25^,^26^,^27^. Additionally, it has been reported that collagen has the innate ability to form magnetically responsive fibrils capable of directing neurite regeneration for nerve repair^27^,^28^. We previously reported the development and implementation of a collagen-based nerve guidance bio-scaffold known as the linear ordered collagen scaffold in SCI^24^,^29^,^30^,^31^. In the present study, this scaffold was renamed the NeuroRegen scaffold and used to provide both directional guidance for axonal growth and as a carrier system for MSCs in a canine model of SCI. Of note, canine SCI not only has comparable clinical signs and a similar poor prognosis with respect to SCI in humans but also provides an alternative biological model to bridge the gap between rodents and humans^32^.

In this study, the NeuroRegen scaffold was functionalized by loading with hUC-MSCs and implanted into the canine spinal cord after complete resection of the T8 spinal cord segment and subsequent glial scar removal. We evaluated therapeutic effects on axonal regeneration, injury site characteristics, and locomotor recovery. Our findings may highlight the clinical value of the functionalized NeuroRegen scaffold for SCI recovery.

## Results

**Figure 1:**
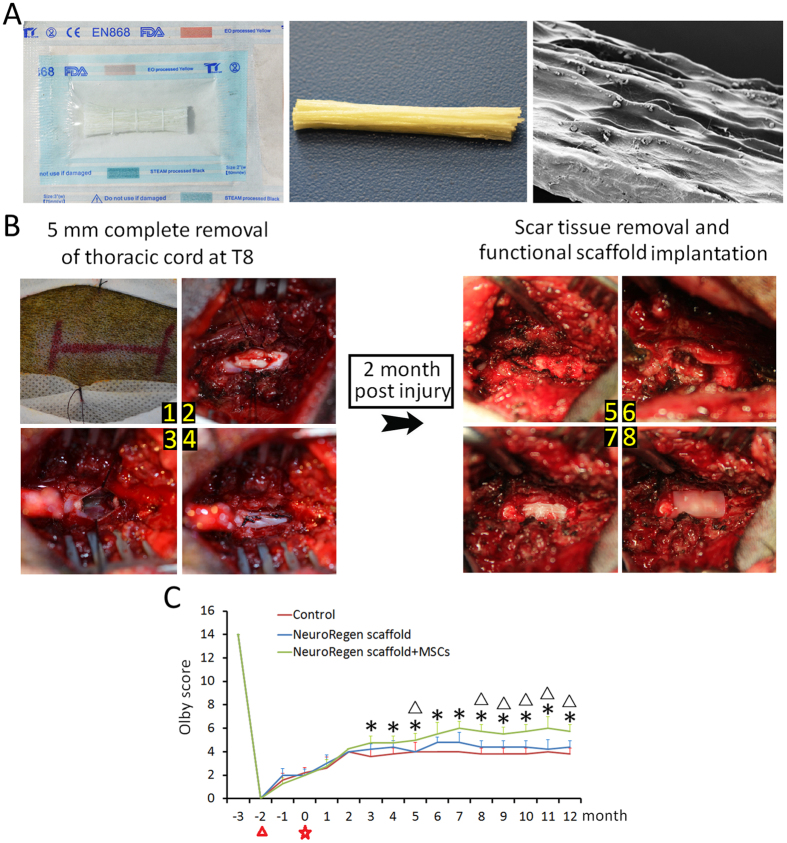
NeuroRegen scaffold implantation procedure. (**A**) An aseptic package, a NeuroRegen scaffold bundle, and a scanning electron micrograph image of a single scaffold fiber. (**B**) Illustration of the canine T8 spinal cord resection model and implantation of the NeuroRegen scaffold loaded with hUC-MSCs at 2 months post-injury. (**C**) Assessment of locomotor recovery with Olby scores at the indicated time points post-injury. Canines in NeuroRegen scaffold with MSCs group showed the greatest locomotor improvement during the observation period. 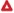 indicates the time point of T8 spinal cord injury; 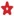 indicates the time point of scar tissue removal; * indicates P < 0.05 compared to the control group; and Δ indicates P < 0.05 compared to non-functionalized NeuroRegen scaffold group.

In a previous study, we observed a striking therapeutic effect after implantation of the NeuroRegen scaffold with bound brain-derived neurotrophic factor (BDNF) in a canine model of complete transection acute SCI^30^. The NeuroRegen scaffold promoted both locomotor recovery and axonal regeneration. Nonetheless, the guidance of regenerating axons to bridge the injury cavity after chronic SCI remains an arduous challenge. Here, we examined the ability of the NeuroRegen scaffold functionalized with hUC-MSCs to promote axonal regeneration, bridging, and locomotor recovery after chronic (2-month delay between injury and implantation) canine SCI. In total, 18 adult female Beagle dogs underwent complete T8 spinal cord transection and then received surgery for scar tissue removal at 2 months post-injury (Fig. 1B). Canines were then divided into 3 groups: a control group, a non-functionalized NeuroRegen scaffold implantation group, and a functionalized (hUC-MSCs) NeuroRegen scaffold implantation group. One year after implantation, canines were sacrificed and spinal cord histological sections were evaluated.

### Functionalized NeuroRegen scaffold transplantation promotes locomotor recovery after chronic SCI

We applied the Olby scoring system to evaluate locomotor recovery in canines after chronic SCI. A baseline was taken 1 month before surgery and the total observation period lasted about 15 months. All canines had Olby scores of 14 (indicating normal function) for the lower limbs before surgery and scores of 0 (indicating no motor function) immediately after surgery (Fig. 1C). Spontaneous locomotor recovery with scores up to 2–3 was observed between the time of injury and that of scar tissue removal (from month −2 to month 0). To our surprise, Olby scores were the same before and after scar tissue removal (i.e., no surgery-related decreases in Olby scores were observed). During the subsequent 1-year observation period, Olby scores of injured dogs in the control group showed slight initial improvements (scores of 2–4), but failed to improve further after 5 months post-implantation. This result indicated a very limited innate capacity for self-healing and locomotor recovery after chronic SCI. Canines in the non-functionalized NeuroRegen scaffold group showed little advantage compared to the control group with Olby scores ranging from 2.5 to 4.5, which plateaued after 4 months post-implantation. Canines in the functionalized NeuroRegen scaffold group showed the best locomotor recovery, achieving Olby scores of approximately 6 (indicating occasional weight-bearing protraction of the lower limbs), which plateaued after 7 months post-injury (Fig. 1C).

### Enhanced lesion site neurogenesis/regeneration at 1 year post-implantation of the functionalized NeuroRegen scaffold

**Figure 2:**
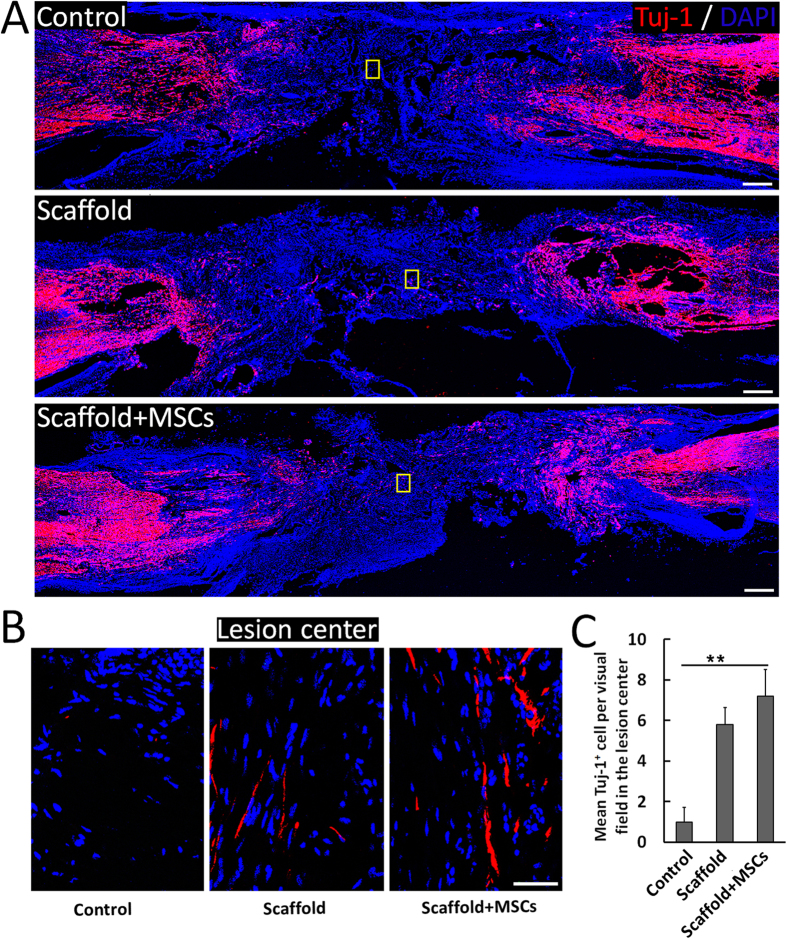
Profiles of newborn neurons after complete T8 resection and glial scar removal. (**A**) At 1 year post-injury, significantly more newborn neurons (Tuj-1+ cells) were detected in the lesion site of canines in NeuroRegen scaffold with MSCs group compared to the control and NeuroRegen scaffold groups. (**B**) Enlarged images of Tuj-1+ cells of the yellow box in the control, NeuroRegen scaffold, and NeuroRegen scaffold with MSCs groups in (**A**). (**C**) Quantification of newborn neurons in the lesion center of canines in each group, respectively. Scale bars represent 1 mm in (**A**) and 50 μm in (**B**). Error bars indicate the standard deviation of 3 different experiments. **Indicates p < 0.01.

**Figure 3:**
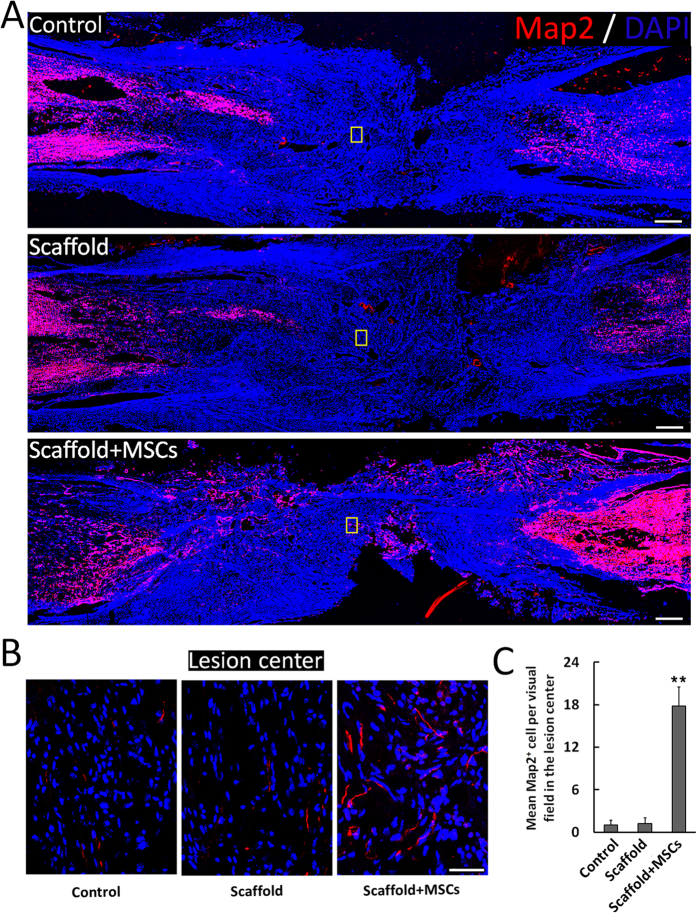
Profiles of mature neurons after complete T8 resection and glial scar removal. (**A**) At 1 year post-injury, significantly more mature neurons (Map2+ cells) were detected in the lesion site of canines in NeuroRegen scaffold with MSCs group compared to the control and NeuroRegen scaffold groups. (**B**) Enlarged images of Map2+ cells of the yellow box in the control, NeuroRegen scaffold, and NeuroRegen scaffold with MSCs groups in (**A**). (**C**) Quantification of mature neurons in the lesion center of canines in each group, respectively. Scale bars represent 1 mm in (**A**) and 50 μm in (**B**). Error bars indicate the standard deviation of 3 different experiments. **Indicates p < 0.01.

Canines were sacrificed at 1 year post-implantation and spinal cord sections were prepared for histology. Samples from the functionalized NeuroRegen scaffold group showed slightly higher numbers of Tuj-1-positive neurons in the lesion area compared to the control and non-functionalized NeuroRegen scaffold groups (Fig. 2). Tuj-1 positive signal was detectable throughout the whole lesion area in functionalized NeuroRegen scaffold groups (Fig. 2B). We also detected higher numbers of Map2-positive mature neurons in the lesion area in samples from the functionalized NeuroRegen scaffold group compared to the control and non-functionalized NeuroRegen scaffold groups (Fig. 3C). In addition, regenerated neurons in the functionalized NeuroRegen scaffold group exhibited long neurite outgrowths that were further indicative of improved regeneration (Fig. 3B).

**Figure 4:**
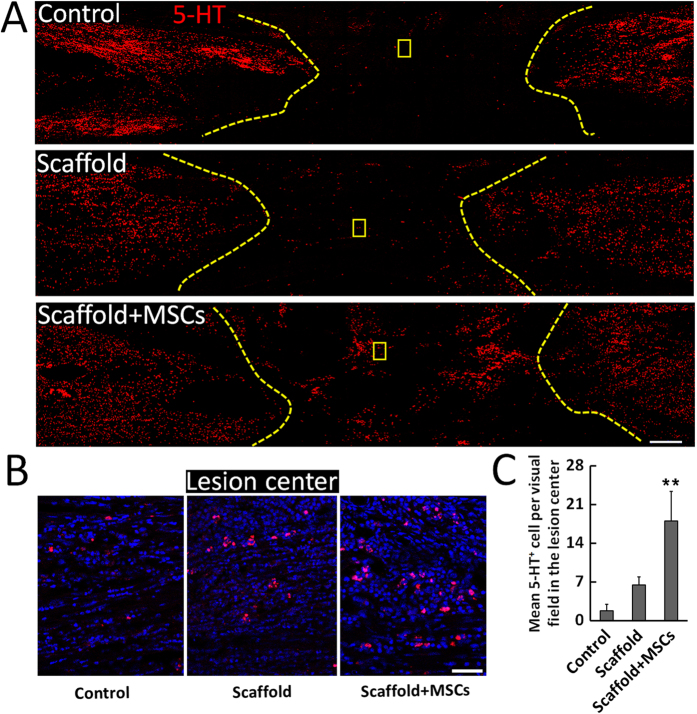
Profiles of 5-HT-positive nerve fibers after complete T8 resection and glial scar removal. (**A**) At 1 year post-injury, 5-HT positive nerve fibers were only detected in the lesion site of canines in NeuroRegen scaffold with MSCs group. (**B**) Enlarged images of 5-HT-positive nerve fibers of the yellow box in the control, NeuroRegen scaffold, and NeuroRegen scaffold with MSCs groups in (**A**). (**C**) Quantification of 5-HT-positive nerve fibers in the lesion center of canines in each group. Scale bars indicate 1 mm in (**A**) and 50 μm in (**B**). Error bars indicate the standard deviation of 3 different experiments. **Indicates p < 0.01.

Based on observations of occasional weight-bearing protraction of the lower limbs and improved lesion site neuronal regeneration in the functionalized NeuroRegen scaffold group, we further evaluated whether regenerated neurons contributed to improved locomotor recovery. Canines in the functionalized NeuroRegen scaffold group exhibited much more 5-HT-positive neurons in the lesion site at 1 year post-implantation than the control and non-functionalized NeuroRegen scaffold groups (Fig. 4). This result indicated that transplantation with the functionalized NeuroRegen scaffold improved the regeneration of 5-HT motoneurons as a cellular foundation for locomotor recovery.

### Remyelinated axons and functional synapses in the lesion center at 1 year post-implantation of the functionalized NeuroRegen scaffold

**Figure 5:**
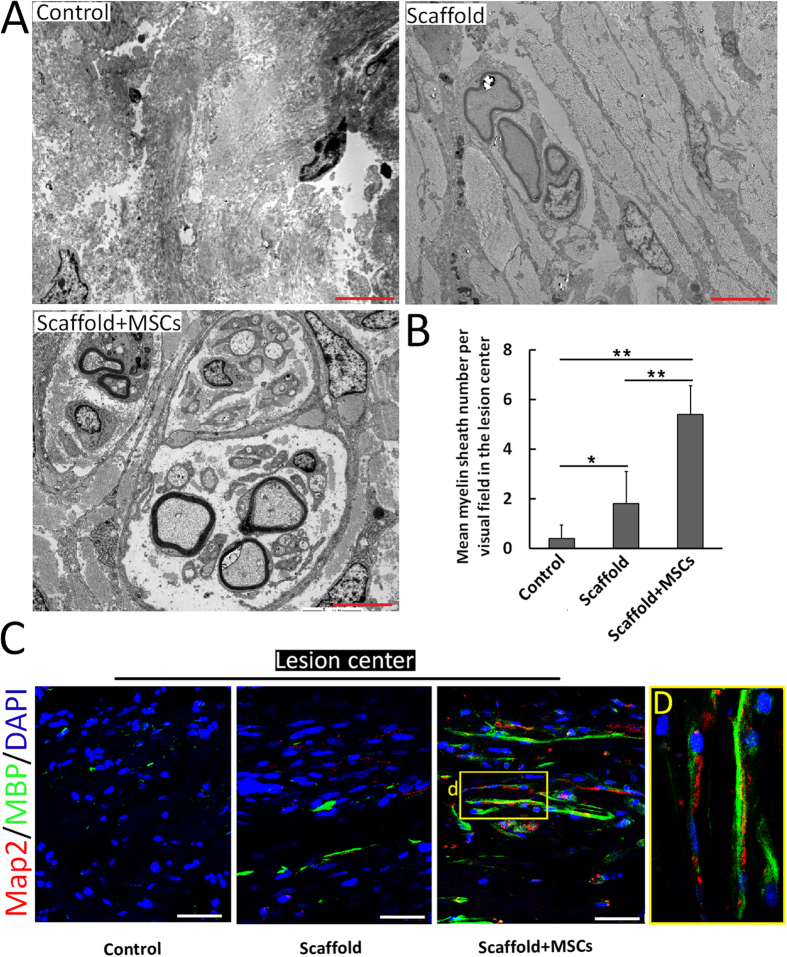
Remyelination of regenerated axons in the lesion site after complete T8 resection and glial scar removal. (**A**) Representative transmission electron microscopy images showing remyelinated nerve fibers in the lesion site of canines in the control, NeuroRegen scaffold, and NeuroRegen scaffold with MSCs groups at 1 year post-injury. Scale bars indicate 5 μm. (**B**) Quantification of newly formed myelin sheath in each group, respectively. Error bars indicate the standard deviation of 3 different experiments. **Indicates p < 0.01. (**C**) Confocal microscopy images double-labeled for Map2 (red) and myelin basic protein (MBP; green) from representative sections in each groups. Scale bars indicate 50 μm. (**D**) Enlarged image of the yellow box in the right panel of (**C**).

Given the observation of Map2-positive and 5-HT-positive neurons in the functionalized NeuroRegen scaffold group, we considered that newly generated neurons might form neuronal relays to bridge the injury site as another cellular basis for locomotor recovery. Thus, we determined whether regenerated axons in the lesion site were remyelinated and formed synapses. Transmission electron microscopy (TEM) showed few remyelinated fibers in the lesion center of control group canines and only scattered remyelination in the non-functionalized NeuroRegen scaffold group. In contrast, remyelinated axons were more numerously detected in the lesion center in the functionalized NeuroRegen scaffold group (Fig. 5A). Quantification of total myelin sheath in the lesion center further confirmed this finding (Fig. 5B). The above result was additionally corroborated by double staining for MBP and Map2; most Map2-positive fibers that projected beyond the lesion border were MBP-positive, indicating that regenerated fibers in the lesion site were probably functionally remyelinated (Fig. 5C). Some Map2-positive fibers within the lesion center were also successfully remyelinated in the functionalized NeuroRegen scaffold group (Fig. 5C,D). These data showed that functionalized NeuroRegen scaffold transplantation not only enhanced axonal growth but also promoted the remyelination of newly regenerated axons.

**Figure 6:**
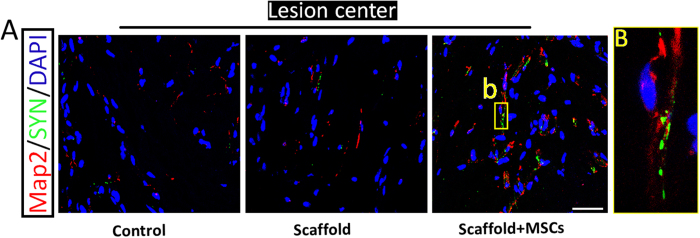
The functionalized NeuroRegen scaffold facilitates synapse formation in the lesion site after complete T8 resection and glial scar removal. (**A**) At 1 year post-injury, confocal microscopy images double-labeled for Map2 (red) and synaptophysin (SYN; green) from representative sections in the lesion site of canines in the control, NeuroRegen scaffold, and NeuroRegen scaffold with MSCs groups. (**B**) Enlarged image of the yellow box in the right panel of (**A**). Scale bars indicate 50 μm.

Finally, to determine whether successfully remyelinated axons formed neural circuits, we investigated the formation of functional synapses in the lesion area. Only canines in the functionalized NeuroRegen scaffold group exhibited appreciable synaptophysin (SYN) signal in the lesion area in the functionalized NeuroRegen scaffold group (Fig. 6A). Additionally, abundant SYN signal was detected near the rostral and caudal lesion borders in all groups (Fig. 6B). These results suggested that improved functional synapse formation in the functionalized NeuroRegen scaffold group might have facilitated locomotor recovery at 1 year post-implantation.

### Reduced secondary glial scar formation in the lesion site at 1 year post-implantation of the NeuroRegen scaffold

**Figure 7:**
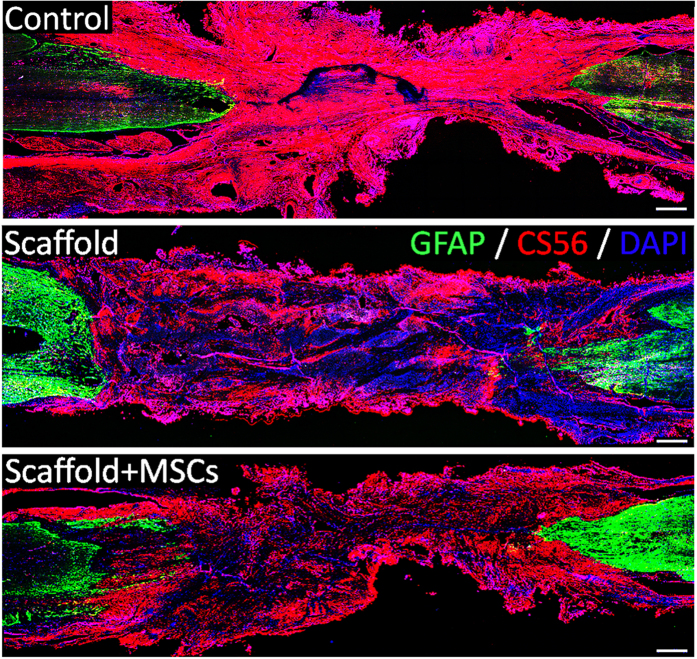
The functionalized NeuroRegen scaffold reduces secondary glial scarring after complete T8 resection and initial glial scar removal. At 1 year post-injury, CS-56 signal as a surrogate of chondroitin sulfate proteoglycan deposition was much weaker in the lesion site of canines in the functionalized and non-functionalized NeuroRegen scaffold groups than in the control group. Scale bars indicate 1 mm.

Studies have shown that glial scarring impedes axonal regeneration, and that transected axons do not project beyond the borders of glial scar barriers^33^. Therefore, reduced glial scar formation is thought to have a stimulatory effect on axonal regeneration and functional recovery. Hence, we surgically resected scar tissue from the lesion area before NeuroRegen scaffold implantation. At 1 year post-implantation, CS-56 signal as a surrogate of CSPG deposition and thus secondary glial scarring was observed throughout the lesion area in the control group (Fig. 7). In contrast, CS-56 signal was lower in the functionalized and non-functionalized NeuroRegen scaffold groups (Fig. 7), suggesting that NeuroRegen scaffold implantation limited the extent of secondary glial scar formation.

## Discussion

A complex and adverse lesion microenvironment after chronic spinal cord injury hampers the ability of treatment to improve locomotor outcomes after SCI. Given their neuroprotective properties, MSCs from various sources have been applied for the treatment of SCI in preclinical and clinical trials^34^. We previous tested the therapeutic utility of our NeuroRegen collagen scaffold system in both rodent and canine models of SCI and observed improved nerve regeneration and functional recovery^24^,^25^,^26^,^27^; in the present study, we combined this technology with MSCs and found that MSC-functionalized NeuroRegen scaffolds also improved nerve regeneration, myelination, and functional recovery after complete T8 resection in canines. Moreover, NeuroRegen scaffold transplantation attenuated secondary glial scarring after initial scar removal. Together, our findings suggest that the neuroprotective properties of transplanted hUC-MSCs in combination with the glial scar-reducing effects of the NeuroRegen scaffold led to motor improvements after SCI.

While MSCs can differentiate into several distinct cell lineages^35^, evidence suggests that MSCs primarily exert therapeutic effects in SCI through immunomodulatory and paracrine mechanisms in the lesion site rather than through direct differentiation into replacement nerve cells^34^,^36^,^37^. Furthermore, transplanted MSCs are not reported to survive long-term in the spinal cord^38^,^39^. Therefore, increased numbers of neurons (both newborn and mature neurons) in the lesion site in the functionalized NeuroRegen scaffold group were probably generated from endogenous neuronal progenitor cells and promoted by the neuroprotective influences of MSCs. Many studies have reported that ependymal cells (ECs) in the central canal of the adult spinal cord serve as neural progenitor cells^40^,^41^. Proliferating ECs are reported to migrate from the central canal towards the injury epicenter within 3 days after SCI^42^. While most studies have indicated that a majority of these ECs differentiate into new astrocytes to form glial scars and few differentiate into neurons^43^,^44^, a recent study illustrated that EC-derived neurons can participate in recovery after SCI in rats^45^. In this study, we observed robust neurogenesis in the lesion site after hUC-MSCs grafting via the NeuroRegen scaffold. Together, it can be hypothesized that increased neuronal generation from intrinsic neural stem cells was facilitated by the microenvironment created in the lesion site by grafted hUC-MSCs. Notably, in this study, we did not investigate the fate of transplanted hUC-MSCs in the early weeks post-transplantation owing to a limited number of canines. However, we stained for human-specific mitochondrial and nuclear markers in spinal cords at 6 months post-transplantation and found no evidence of human cells in lesion sites or in intact spinal tissue.

Neurogenesis in the lesion site alone was insufficient to indicate an association with improved locomotor outcomes in the functionalized NeuroRegen scaffold group; thus, we confirmed the maturation of newly generated neurons into 5-HT-positive neurons in the lesion area. These neurons may account for the observation of occasional weight-bearing limb protraction in canines from the functionalized NeuroRegen scaffold group. Additionally, remyelination of regenerated axons in the lesion area in the functionalized NeuroRegen group was confirmed using both TEM and MBP/Map2 double-immunofluorescence, suggesting that improved remyelination may have also led to better locomotor outcomes. Lastly, functional synapses (SYN/Map2 double staining) were uniquely observed in the lesion site of canines in the NeuroRegen scaffold group, further providing a cellular basis for improved locomotor recovery after functionalized NeuroRegen scaffold implantation. Together, the application of hUC-MSCs via the NeuroRegen scaffold appears to mitigate the adverse microenvironment of SCI lesions, leading to enhanced neurogenesis, remyelination, and synapse formation as cellular foundations for locomotor recovery. Finally, astrocyte activation after SCI is thought to be beneficial for limiting the extent of secondary injury related to tissue damage; however, excessive astrogliosis leads to glial scar formation and produces a physical barrier to axonal regeneration^46^. In addition, inhibitory molecules secreted by glial scar tissue such as CSPGs act as a biochemical barrier to impede the reestablishment of neural circuitry after injury. In this study, glial scar tissue was removed before implantation to facilitate recovery. Notably, we observed that canine Olby scores did not decrease to 0 after scar tissue removal; rather, these scores were unchanged post-surgery. This indicated that self-repair and locomotor recovery were not hampered by the resection of glial scar tissue (and was independent of the penetration of host axons); additionally, scar tissue resection before functionalized NeuroRegen scaffold implantation was safe and did not lead to locomotor retrogression in canines. A similar phenomenon was observed by Lu *et al*. in a rodent SCI model; mean locomotor scores (BBB at score 2) were not altered after re-transection in the control group, and scores up to 7 were achieved after re-transection in a neural stem cell transplantation group^47^. Unlike previous study has reported that the improperly tuned mechanical properties of the collagen scaffold might act as a physical barrier for regenerating axon fibers^48^. At 1 year post-transplantation in our study, dense CSPG-positive signal was detected throughout the lesion site in control canines, indicating the development of secondary scarring. Notably, this secondary scarring was largely diminished in canines from the functionalized and non-functionalized NeuroRegen scaffold groups. Decreased CS-56 deposition independent of hUC-MSC transplantation suggested that the NeuroRegen scaffold itself inhibited secondary glial scarring. Thus, it can be concluded that the combined effects of transplanted hUC-MSCs and the ability of the NeuroRegen scaffold to inhibit glial scarring collectively promoted functional repair and locomotor recovery after chronic SCI. Future studies are required to evaluate the potential utility of the functionalized NeuroRegen apparatus in clinical settings.

## Methods

### Ethics statement

Animal experiments were performed in accordance with Guide for the Care and Use of Laboratory Animals from National Institutes of Health and approved by the Animal Care and Use Committee of Xiangya Hospital, Central South University in Hunan Province of China. All animal procedures were in accordance with the National Institute of Health’s Guide for the Care and Use of Laboratory Animals. Five human umbilical cords were obtained from healthy newborns, and the obtained umbilical cords for research have got the informed consent of the newborns’ parents and their family members. The study were reviewed and approved by the Ethics Committee of Xiangya Hospital. All methods were performed in accordance with the relevant guidelines and regulations of the Animal Care and Use Committee and the Ethics Committee of Xiangya Hospital.

### Isolation and culture of hUC-MSCs

The umbilical cord tissue was washed in a Petri dish containing 40 mL sodium chloride with sterile forceps to remove blood. After the blood vessels and amnion were removed, Wharton’s jelly was collected in a new Petri dish. The Wharton’s jelly was then sliced into small pieces with 1 mm in diameter before transferred to culture flasks which were covered with glass slides to prevent from floating. After adhered to the flasks, the hUC-MSCs were cultured in serum-free MesenCult-XF medium and FBS bases DMEM complete medium (Stemcell, Vancouver, Canada) at 37 °C in a humidified atmosphere with 5% CO_2._ Additionally, the medium was changed every 3–4 days. hUC-MSCs were digested with 0.05% trypsin/EDTA before passage. The morphology of hUC-MSCs were observed and recorded under a microscope.

### The preparation of NeuroRegen scaffold

NeuroRegen scaffold was prepared from bovine aponeurosis as described before^49^. Aponeuroses of 0.5 mm thickness were separated from muscles, and then were cut into the proper size. The adjunctive tissues of aponeurosis, including the residual muscles, connective tissues and fats were further removed. Figure 1A showed an aseptic package and a bundle of NeuroRegen scaffold to be transplanted and the scanning electron micrograph of a single fiber.

### Surgery procedures, postoperative care and rehabilitation

Surgery process was similar as we described before with some modification^30^,^31^. A total of 18 adult female Beagle dogs (Dasuo Biotechnology Inc. sichuang, China) were normally housed in temperature and humidity controlled laboratory animal room at Animal Department of Xiangya Medical school. The dogswere anesthetized by subcutaneous administration of Xylazine hydrochloride 8 mg/kg and ketamine at 2.5 mg/kg after 8–10 hours of preoperative fasting and water deprivation. The hair on the back of the anesthetized dog was shaved and the skin was cleaned with povidone iodine. Operation was performed under the sterile conditions. Physiological saline was used to compensate for body fluid loss during operation. A 5–6 centimeter longitudinal incision was made through the skin above the 8th thoracic vertebra. The back musculature was separated along spinous process. A dorsal laminectomy was performed at the level of T8 using bone rongeur. A longitudinal incision was made through the dura, exposing 1.5 cm of spinal cord. a 5 mm of spinal cord was completely removed by microscissors. Gelfoam was temporarily placed in the gap for hemostasis, then the incision was sutured layer by layer. The dogs were housed in laboratory animal room with suitable temperature and humidity. Antibiotics and glucose intravenously was adopted for 3 days. Dogs lacked normal micturition reflex and their bladders were manipulated quartic a day.

Two months after surgeon, dogs in chronic spinal cord injury phase were received anesthesia induction by subcutaneous administration of Xylazine hydrochloride (8 mg/kg) and ketamine (2.5 mg/kg) after 8–10 hours of preoperative fasting and water deprivation. After successful establishment of endotracheal intubation and intravenous, anesthesia was maintained by propofol and fentanyl. Physiological saline was used to compensate for body fuild loss during surgery. The primary longitudinal incision was adopted and the tissue was separated though monopolar electrical cautery to expose the spinous process and canalis vertebralis. Both of the scar tissues deposit outside and within the spinal dura mater were totally removed by microscissors under microscope (zeiss). The functional collagen scaffold, 1.2–1.5 mm long and 5 mm diameter bundle of NeuroRegen scaffold alone or loaded with 10^7^ hUC-MSCs, were grafted into the scar tissue removed gap to bridge the defects. The collagen membrane was placed over the exposed cord and sutured to the cut edges of the dura to prevent the peridural adhesion and scar formation.

According to the implants between the transected sites, all animals were allocated into three groups: Control group (n = 6); NeuroRegen scaffold implantation group (n = 6) and NeuroRegen scaffold loaded with hUCMSCs transplantation group (n = 6).

After the operation, each dog routinely received antibiotics and glucose intravenously for 5 days and its bladder had to be emptied manually every 12 h for 2 weeks due to a lack of normal micturition reflexes. The rehabilitation was initiated 3 weeks after surgery. The multisystem rehabilitation and exercise training was as previously described^31^.

### Behavioral assessment

We used Olby scores to evaluate functional motor recovery in canine SCI^50^. Two individuals unaware of experimental conditions recorded an Olby score for each dog at months 1, 2 after the transection surgery and months 1 to 12 after treatments. During the assessment, the dogs moved freely in an open field and were rated on the basis of their ability for spontaneous or voluntary hindlimb motion. The detailed scoring rules of the Olby score system was provided in Table 1.

### Histological analysis

At week 38 after operation, the spinal cord from T5-T11 was retrieved and fixed in 4% (v/v) formaldehyde for 48 h. The segments were then embedded in paraffin and cut into 10-μm thick sections (Leica Microsystems).

For immunofluorescence staining, the paraffin embedded sections were subjected to heat mediated antigen retrieval using citric acid, and the sections were then blocked using normal horse serum/T-PBS (1:20) for 15 min. Primary antibodies were applied to the sections and incubated overnight at 4 °C. The primary antibodies were against the following: GFAP (1:800, ab7779, Abcam), 5-HT (1:800, 20080, Immuostar), CS-56 (1: 500, ab11570, Abcam), Myelin Basic Protein (1: 500, ab24567, Abcam), Tuj-1/beta III Tubulin (1: 500, ab7751, Abcam), Map2 (1:500, M1406, Sigma), and SYN (1: 500, Ls-c174790, LSBio). Sections were then incubated with secondary antibody (Alexa Fluor 488, 1:400; Alexa Fluor 594, 1:800; Invitrogen). Cell nuclei were stained with DAPI (1:1000, sigma) and images were taken under the Leica TCS SP8 Confocal Microscope (Leica Microsystems, Germany). Image-Pro Plus software (Media Cybernetics LP, Maryland, USA) was used to quantify immunostaining positive signals by selecting at least 3 fields per sample at the lesion center.

### Transmission electron microscope (TEM) analysis

Transmission electron microscopic analysis was carried out according to the previous protocol^30^. Briefly, spinal cord samples of canines were cut into small pieces and fixed with 2.5% glutaraldehyde solution at 4 °C. By washing with 0.1 M phosphate buffer (PB, pH 7.2) for three times, the specimens were post fixed with 1% OsO4 for 2 h. After additionally washed three times by 0.1 M PB, the specimens were dehydrated in ascending gradual series (50e100%) of acetone. Then the specimens were embedded by Poly/Bed 812 kit (Polysciences). After polymerized at 60 °C for 24 h, the specimens were were cut into 70 nm ultra-thin sections by Leica Ultracut UCT Ultramicrotome (Leica Microsystems). The sections were double stained with uranyl acetate and lead citrate. All of the thin sections were observed under transmission electron microscope JEM 1400.

### Statistical analysis

Data were presented as mean values ± standard deviation. SPSS 13.0 was used for all statistical analysis. Olby scores for each group were analyzed using repeated measures ANOVA. Comparisons of quantitative immunohistochemistry data were performed with ANOVA. P values < 0.05 were considered significant.

## Additional Information

**How to cite this article**: Li, X. *et al*. Transplantation of hUC-MSCs seeded collagen scaffolds reduces scar formation and promotes functional recovery in canines with chronic spinal cord injury. *Sci. Rep.* **7**, 43559; doi: 10.1038/srep43559 (2017).

**Publisher's note:** Springer Nature remains neutral with regard to jurisdictional claims in published maps and institutional affiliations.

## Figures and Tables

**Table 1:** Assessment of pelvic limb function by Olby score^50^.

Stage	Point	Neurological status
1	0	No pelvic limb movement and no deep pain sensation.
	1	No pelvic limb movement with deep pain sensation.
	2	No pelvic limb movement but voluntary tail movement.
2	3	Minimal non-weight-bearing protraction of pelvic limb (movement of one joint).
	4	Non-weight-bearing protraction of pelvic limb with more than one joint involved less than 50% of the time.
	5	Non-weight-bearing protraction of pelvic limb with more than one joint involved more than 50% of the time.
3	6	Weight-bearing protraction of pelvic limb less than 10% of the time.
	7	Weight-bearing protraction of pelvic limb 10–50% of the time.
	8	Weight-bearing protraction of pelvic limb more than 50% of the time.
4	9	Weight-bearing protraction 100% of time with reduced strength of pelvic limb. Mistake >90% of the rime.
	10	Weight-bearing protraction of pelvic limb 100% of time with reduced strength. Mistake 50% - 90% of the rime.
	11	Weight-bearing protraction of pelvic limb 100% of time with reduced strength. Mistake <50% of the rime.
5	12	Ataxic pelvic limb gait with normal strength, but mistakes made >50% of time.
	13	Ataxic pelvic limb gait with normal strength, but mistakes made <50% of time.
	14	Normal pelvic limb gait.
